# 2-Methoxyestradiol-3,17-*O,O*-bis-sulfamate inhibits store-operated Ca^2+^ entry in T lymphocytes and prevents experimental autoimmune encephalomyelitis

**DOI:** 10.1016/j.bbamcr.2023.119485

**Published:** 2023-05-06

**Authors:** Leon Hosang, Anke Löhndorf, Wolfgang Dohle, Anette Rosche, Stephen Marry, Björn-Philipp Diercks, Lukas C. Müller-Kirschbaum, Lioba T. Flügel, Barry V.L. Potter, Francesca Odoardi, Andreas H. Guse, Alexander Flügel

**Affiliations:** 1 Institute for Neuroimmunology and Multiple Sclerosis Research, University Medical Center Göttingen, Von-Siebold-Straße 3a, D-37075 Göttingen, Germany; 2 The Calcium Signalling Group, Department of Biochemistry and Molecular Cell Biology, University Medical Center Hamburg-Eppendorf, Martinistraße 52, D-20246 Hamburg, Germany; 3 Drug Discovery & Medicinal Chemistry, Department of Pharmacology, University of Oxford, Mansfield Road, Oxford OX1 3QT, United Kingdom; 4 Department of Neurology, University Medical Center Göttingen, Robert-Koch-Straße 40, D-37075 Göttingen, Germany

## Abstract

Ca^2+^ signaling is one of the essential signaling systems for T lymphocyte activation, the latter being an essential step in the pathogenesis of autoimmune diseases such as multiple sclerosis (MS). Store-operated Ca^2+^ entry (SOCE) ensures long lasting Ca^2+^ signaling and is of utmost importance for major downstream T lymphocyte activation steps, e.g. nuclear localization of the transcription factor ‘nuclear factor of activated T cells’ (NFAT). 2-Methoxyestradiol (2ME2), an endogenous metabolite of estradiol (E2), blocks nuclear translocation of NFAT. The likely underlying mechanism is inhibition of SOCE, as shown for its synthetic sulfamate ester analogue 2-ethyl-3-sulfamoyloxy-17β-cyanomethylestra-1,3,5(10)-triene (STX564).

Here, we demonstrate that another synthetic bis-sulfamoylated 2ME2 derivative, 2-methoxyestradiol-3,17-*O,O*-bis-sulfamate (2-MeOE2bisMATE, STX140), an orally bioavailable, multi-targeting anticancer agent and potent steroid sulfatase (STS) inhibitor, antagonized SOCE in T lymphocytes. Downstream events, e.g. secretion of the pro-inflammatory cytokines interferon-γ and interleukin-17, were decreased by STX140 in *in vitro* experiments.

Remarkably, STX140 dosed *in vivo* completely blocked the clinical disease in both active and transfer experimental autoimmune encephalomyelitis (EAE) in Lewis rats, a T cell-mediated animal model for MS, at a dose of 10 mg/kg/day i.p., whereas neither 2ME2 nor Irosustat, a pure STS inhibitor, showed any effect. The STS inhibitory activity of STX140 is therefore not responsible for its activity in this model.

Taken together, inhibition of SOCE by STX140 resulting in full antagonism of clinical symptoms in EAE in the Lewis rat, paired with the known excellent bioavailability and pharmaceutical profile of this drug, open potentially new therapeutic avenues for the treatment of MS.

## Abbreviations

[Ca^2+^]_i_: free cytosolic and nuclear calcium concentration
2ME2: 2-methoxyestradiol
ADP: adenosine 5’-diphosphate
APC: antigen presenting cell
CD: cluster of differentiation
CFA: complete Freund’s Adjuvant
CNS: central nervous system
DMPK: drug metabolism and pharmacokinetics
DMSO: dimethyl sulfoxide
E2: estradiol
EMATE: estrone-3-*O*-sulfamate
EAE: experimental autoimmune encephalomyelitis
GFP: green fluorescent protein
G-GECO: green fluorescent genetically encoded Ca^2+^ indicator for optical imaging
IFNγ: interferon-γ
IgG: immunoglobulin G
IL: interleukin
IP_3_: D-*myo*-inositol 1,4,5-trisphosphate
MFI: mean fluorescent intensity
MBP: myelin basic protein
MS: multiple sclerosis
NAADP: nicotinic acid adenine dinucleotide phosphate
NCD: NanoCrystal dispersion
NCS: newborn calf serum
NFAT: nuclear factor of activated T cells
PBMCs: peripheral blood mononuclear cells
qPCR: quantitative polymerase chain reaction
S1PR1: sphingosine-1-phosphate receptor 1
SOCE: store-operated Ca^2+^ entry
STX140: 2-methoxyestradiol-3,17-*O,O*-bis-sulfamate
STX564: 2-ethyl-3-sulfamoyloxy-17β-cyanomethylestra-1,3,5(10)-triene
TCR: T cell receptor
TG: tapsigargin
Th1/17: T helper cells type 1/17
T_MBP_: myelin basic protein-reactive CD4^+^ rat T cells
TNFα: tumour necrosis factor-α
VLA4: very late antigen-4
WT: wild type.

## Introduction

Elevation of the free cytosolic and nuclear Ca^2+^ concentration ([Ca^2+^]_i_) is an essential event during T cell activation. It starts with Ca^2+^ release from intracellular Ca^2+^ stores sequentially involving the Ca^2+^ mobilizing 2^nd^ messengers nicotinic acid adenine dinucleotide phosphate (NAADP; reviewed in Guse 2022a, 2022b), D-*myo*-inositol 1,4,5-trisphosphate (IP3; Guse *et al*., 1993) and cyclic ADP-ribose (Guse *et al*., 1999). Ca^2+^ store depletion in turn activates Ca^2+^ entry from the extracellular space, termed store-operated Ca^2+^ entry (SOCE). The major Ca^2+^ channel that operates SOCE in T cells is Orai1 (Feske *et al*., 2007; Peinelt *et al*., 2006; Vig *et al*., 2006; Yeromin *et al*., 2006). Pharmacological or genetic blockade of Orai1 blocks T cell Ca^2+^ signaling and interferes with T cell-mediated autoimmunity in models of multiple sclerosis (MS), colitis and graft versus host disease via interference with the function of the pathogenic Th1/17 cells (Feske *et al*., 2007). Thus, Orai1 and other components of SOCE, e.g. stromal interaction molecules 1 and 2 (STIM1 and 2) represent interesting therapeutic targets for T cell-mediated (auto)immune diseases.

2-Methoxyestradiol (2ME2) is an endogenous metabolite of estradiol (E2, reviewed by Zhu & Conney, 1998) that binds to isolated estrogen receptors with an approximately 500-fold reduced affinity (LaVallee *et al*., 2002). Due to its anti-proliferative and anti-angiogenic properties, 2ME2 has been evaluated for the treatment of several tumour types in human clinical trials, but not without difficulties in bioavailability (Potter, 2018). Mechanistically, 2ME2 inhibits the polymerization of tubulin and the binding of colchicine to it. Thereby, rapidly dividing cells are blocked in the G2/M phase of the cell cycle and undergo apoptosis. Quiescent cells are spared. Moreover, 2ME2 also has anti-inflammatory properties. It ameliorated disease severity in two different models of T cell-mediated autoimmunity, namely collagen-induced arthritis (CIA), a model of rheumatoid arthritis, and experimental autoimmune encephalomyelitis (EAE), a model of MS (Stubelius *et al*., 2011; Duncan *et al*., 2012). The latter study revealed that 2ME2 inhibits T cell activation by impairing the translocation of the transcription factor ‘nuclear factor of activated T cells’ (NFAT) into the nucleus of T cells. More recently, it was shown that 2ME2 and its analogue 2-ethyl-3-sulfamoyloxy-17β-cyanomethylestra-1,3,5(10)-triene (STX564) inhibit SOCE through Orai1 channels (Löhndorf *et al*., 2021). Thus, inhibition of Ca^2+^ entry is likely to be the mechanism underlying 2ME2-mediated inhibition of NFAT translocation, T cell proliferation and cytokine production, as well as G2/M phase cell cycle arrest (Duncan *et al*., 2012; Löhndorf *et al*., 2021).

**Figure 1:**
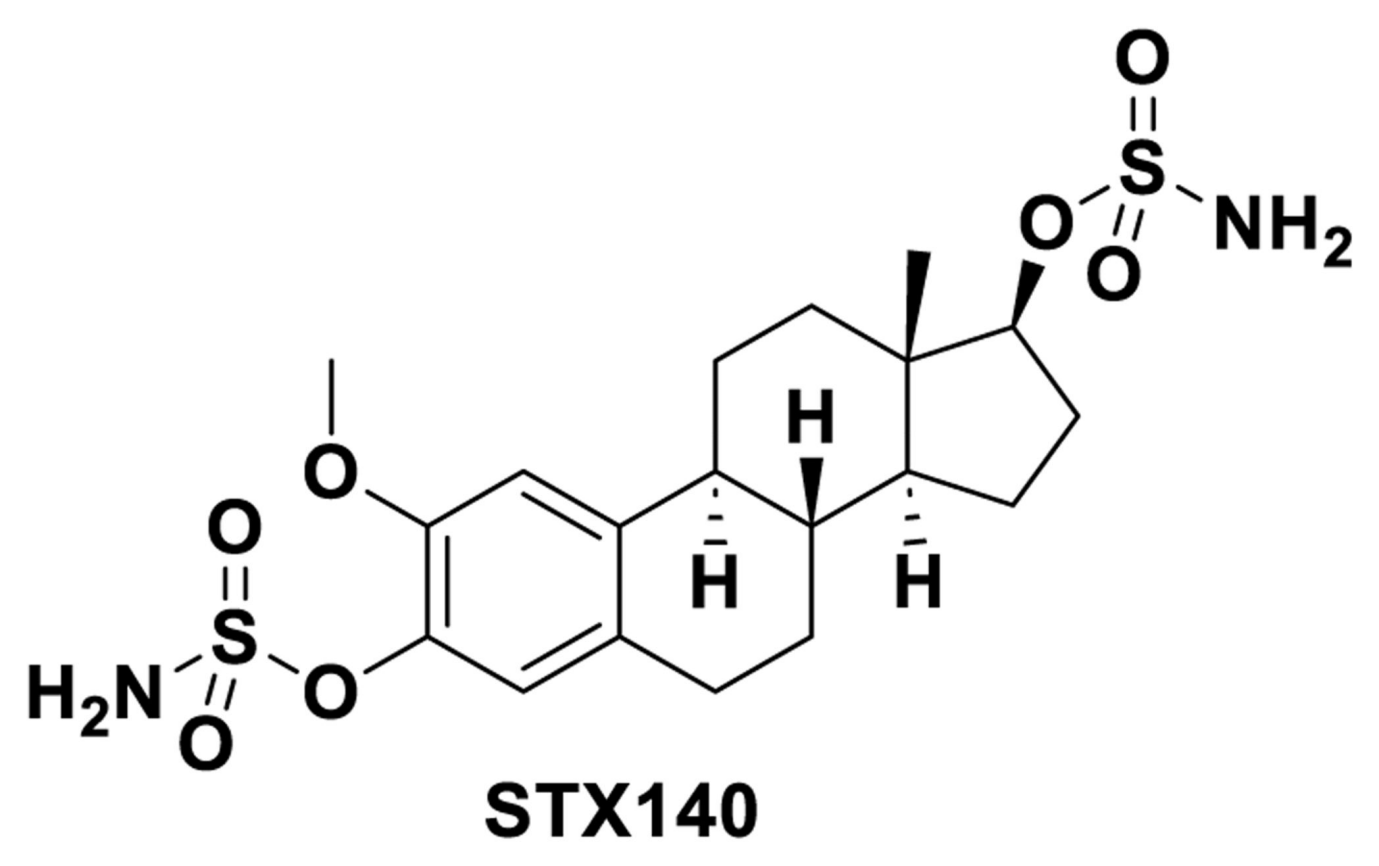
Chemical structure of STX140.

The therapeutic potential of 2ME2 is extremely limited by its poor oral bioavailability of only ~1.5 %. In an approach to solve this issue, 2ME2 was formulated as NanoCrystal dispersion resulting in an increase of the bioavailability to ~3-4 % (Tevaarwerk *et al* 2009). As an alternative approach, numerous 2-substituted estrogen derivatives were synthesized. One of these compounds, 2-methoxyestradiol-3,17-O,O-bis-sulfamate (2-MeOE2bisMATE, STX140; Fig. 1) (Leese et al, 2006), was designed as part of a program exploring non-estrogenic steroid-based inhibitors against the novel oncology target steroid sulfatase (STS) (Reed *et al* 2005; Thomas & Potter, 2015a,b; Potter, 2018). STX140 displayed a very good oral bioavailability (85 %), while retaining multi-targeting and antimitotic properties together with potent STS inhibition (Newman *et al*., 2006, 2007, 2008). Sulfamoylation is known to imbue positive pharmaceutical and DMPK properties, through blockade of metabolism and facilitation of drug sequestration into red blood cells (Ireson et al. 2004a; Potter 2018) through binding to carbonic anhydase II (Ho et al, 2003; Lloyd et al., 2015; Cozier et al 2010;). Moreover, a sulfamate substitution has been shown using crystallography to enhance binding to tubulin (Dohle *et al*., 2018). Whether STX140 also maintains the anti-inflammatory properties of 2ME2, however, had not yet been addressed. Moreover, there is evidence that STS inhibition alone can have anti-inflammatory effects (Reed et al., 2005), so there was also the possibility of synergistic effects using STX140, especially given that in a collagen-induced arthritis model evidence was obtained showing that progression was markedly altered by the STS inhibitor estrone 3-*O*-sulfamate (Foulkes et al 1997).

In this work, we therefore characterized STX140 regarding its potential to inhibit Ca^2+^ entry in T cells and downstream signaling events, e.g. secretion of pro-inflammatory cytokines. Furthermore, we investigated its therapeutic potential in EAE and explored a potential role for STS inhibition using the clinical drug Irosustat (Stanway et al 2006; Potter, 2018).

## Material & Methods

### Animals

Wild-type (WT) rats on a Lewis (LEW/Crl; Rattus norvegicus) genetic background were bred in the animal facilities of the University Medical Center Göttingen (Germany) and held under standardized conditions at a 12/12-hour light/dark cycle with food and water provided ad libitum. All animal experiments were approved by the responsible authorities (number: 33.9-42502-04-19/3250). Male and female animals between 6–12 weeks old were used in the EAE experiments. No differences between the sexes were noted.

### Reagents, drugs and antigen

HEPES was purchased from Roth. 2ME2 was purchased from Tetrionics (Madison, WI, USA). STX140 was synthesized as described in Leese *et al*., 2006 (compound 21). Irosustat was synthesized as described in Woo *et al*., 2000 (compound 17). Unless otherwise stated, chemicals were purchased either from Merck or Sigma. For *in vitro* experiments, 2ME2, STX140 and Irosustat were dissolved in DMSO at 50 mM and stored in single-use aliquots at -20 °C for up to 6 months. Just before the measurements, this stock solution was diluted in DMSO to obtain a DMSO concentration of 0.2 % *(v/v)* for all concentrations measured. Therefore, 0.2 % (*v/v*) DMSO acts as the vehicle control in all *in vitro* experiments. For *in vivo* experiments, STX140 was dissolved in a vehicle, namely 40 % 2-hydroxypropyl-β-cyclodextrin (PanReac AppliChem). Vehicle treatment alone served as control. Myelin basic protein (MBP) was extracted from guinea pig brain as described (Eylar *et al*., 1974, Määttä *et al*., 1997).

### Generation and culturing of T_MBP_ cells

CD4^+^ T cells reactive against MBP and retrovirally transduced to express enhanced GFP (T_MBP_ cells) were generated as previously described (Flügel *et al*., 1999). In brief, 6-8 week old female Lewis rats were immunized subcutaneously with 150 μL of an emulsion consisting to equal parts of MBP (1 mg/mL) and complete Freund’s adjuvant (CFA) containing inactivated *Mycobacterium tuberculosis* extract (Difco; 2 mg/ml). 9-10 days after immunization, the draining lymph nodes were retrieved and passed through a cell strainer (40 μm) to obtain a single cell suspension. The lymph node-derived cells were cultured together with GP+E86 packaging cell lines producing replication-deficient retroviruses encoding for fluorescent proteins in the presence of 10 μg/mL MBP. Between day 2 and 4 after antigen encounter, murine IL-2 was added to the culture to boost T cell expansion. In total, the T cell lines underwent 3-4 cycles of stimulation before being used in both *in vitro* and *in vivo* experiments. All the T cells lines were αβTCR^+^, CD4^+^, CD8^–^, and displayed an effector memory phenotype (L-selectin^-^, CD45RC^low^, CD44^high^). Upon stimulation, they expressed IFNγ and IL-17.

### In vitro activation assay of T_MBP_ cells

To assess T cell activation *in vitro*, 10^5^ T_MBP_ cells and 10^6^ 30 Gy-irradiated thymocytes per well were co-cultured in presence of 10 μg/mL MBP and varying concentrations (0.01, 0.1, 1, 10 μM) of STX140 or the STS inhibitor Irosustat dissolved in DMSO. The assays were performed in flat-bottom 96-well plates. The working concentration of DMSO/well was 0.2 %. As a read-out of T cell activation, the expression of inflammatory cytokine was determined at the mRNA level using quantitative PCR. Furthermore, the expression of surface activation markers was detected at protein levels in T cells by antibody staining and subsequent flow cytometry analysis.

### In vitro activation assay of human PBMCs

*In vitro* activation assays were performed in 96-well plates. Briefly, 10^5^ PBMCs isolated from human peripheral blood by Ficoll gradient were added onto plates coated with anti-human CD3 monoclonal antibody (5 μg mL^–1^ in PBS, clone OKT3, Biolegend). STX140 dissolved in DMSO was added in varying concentrations (0.1, 1, 10 μM). The working concentration of DMSO/well was 0.02% (v/v). PBMC activation on mRNA level was determined using qPCR on samples collected 24 h after CD3 stimulation.

### Culturing of Jurkat T lymphocytes

Jurkat T lymphocytes were cultured as described previously (Löhndorf *et al*., 2021). Briefly, the cells were cultured in RPMI 1640 medium containing 25 mM HEPES and GlutaMAX-1 (Gibco, Life Technologies). The medium was supplemented with 7.5 % *(v/v)* newborn calf serum (NCS, Biochrom, Merck Millipore), 100 U/mL penicillin and streptomycin (standard JMP medium). Cell density was adjusted every two to three days (between 0.3 and 1.3 x 10^6^ cells/mL).

### Fura2 loading

T lymphocytes were loaded with Fura2-AM as described before (Löhndorf *et al*., 2021). In brief, cells were pelleted (Jurkat T lymphocytes: 450 xg, 5 min; rat T_MBP_ lymphocytes: 300 xg, 8 min) at room temperature and were taken up in 1 mL medium (Jurkat T lymphocytes: standard JMP medium; rat T_MBP_ lymphocytes: standard DMEM-based medium containing 10 % (*v/v*) newborn calf serum). Cells were incubated in the dark for 15 min at 37 °C with Fura2-AM (4 μM). Subsequently, 4 mL medium was added, and the cells were incubated in the dark for another 15 min at 37 °C. Afterwards, the cells were washed in 5 mL Ca^2+^ buffer (140 mM NaCl, 5 mM KCl, 1 mM MgSO_4_, 1 mM CaCl_2_, 1 mM NaH_2_PO_4_, 20 mM HEPES, 5.5 mM glucose; pH 7.4 (NaOH), sterile filtered). Then the Jurkat T lymphocytes were diluted to a concentration of 2*10^6^ cells/mL. Cells were kept at room temperature for 20 min to de-esterify before the start of the first measurement.

### Ca^2+^ measurements via fluorescence spectrophotometry and data analysis

10^6^ Fura2-loaded Jurkat T lymphocytes or rat T_MBP_ lymphocytes were pelleted as described above, re-suspended in 1 mL of Ca^2+^ buffer or nominally Ca^2+^ free buffer (as Ca^2+^ buffer but without the addition of 1 mM CaCl2) and were placed in a 10 x 10 mm quartz glass cuvette (Hellma Analytics). The measurements were performed by a fluorescence spectrophotometer (F-2710, Hitachi) and controlled by FL Solutions (version 4.1, Hitachi High-Technologies Corporation) as previously described (Löhndorf *et al*., 2021). Briefly, the probe was alternatively excited at 340 ± 5 nm and 380 ± 5 nm, each 400 ms apart. For Mn^2+^ quenching, the probe was additionally excited at Fura2’s isosbestic point that was experimentally determined to be 358 or 359 nm, depending on the specific instrument. Fluorescence emission was measured at 495 ± 5 nm each. At the end of every Ca^2+^ measurement, with the exception of Mn^2+^ quenching experiments, 0.1 % (*v/v*) Triton X-100 as well as 4 mM EGTA and 30 mM Tris were added to the cells to calibrate the measurement to the maximal and minimal ratio. The free cytosolic Ca^2+^ concentration ([Ca^2+^]_i_) was calculated from the ratiometric data and fluorescent data from the calibration as described in Grynkiewicz *et al*., 1985. Furthermore, artefacts due to compound addition were deleted from the tracings using FL Solutions and deleted data points were interpolated.

ΔPeak values were calculated as the maximum within the first 180 s after the compound addition subtracted by the mean basal value before the first addition. ΔPlateau values were calculated as the mean values between 75 s and 25 s before the next addition or end of the measurement subtracted by the basal values before addition of any compound. For concentration-response curves Δplateau values were normalized to the DMSO control as 100 % and to 19.11 nM [Ca^2+^]_i_ as 0 % as this was the lowest Δplateau value measured at 50 μM of STX49 (Löhndorf *et al*., 2021). For analysing the fast inhibition effect, the minimum value after DMSO or STX140 addition was calculated. This minimum was divided by the plateau value after thapsigargin addition as the mean value between 75 s and 25 s before DMSO or STX140 addition. Then, these data were fitted to the DMSO control as 100 % and to the value of 75 μM STX140 as 0 % as this was the lowest value. Subsequently, the STX140 concentrations were log-transformed, and the data were fitted to a nonlinear fit of variable slope using Prism 9 (GraphPad Software). For Mn^2+^ quenching, the velocity of quenching was calculated as the initial slope after Mn^2+^ addition. First, the fluorescence just before Mn^2+^ addition was set to 1 to better compare different measurements. Then, the data 200 s before Mn^2+^ addition was fitted to a line to avoid overestimation of the initial slope by bleaching using the following equation:

(1) $$y_{lin}=m\cdot x+c$$

*m* is the slope, *x* is the time and *c* is the y-intercept.

Next, the data between 10 s after Mn^2+^ addition and the end of the measurement was fitted to a nonlinear one phase decay using the following equation:

(2) $$y={\left(y_{0}-P\right)}^{\left(-K\cdot X\right)}+P$$

*yo* is the y value at the time point of Mn^2+^ addition. *P* as the plateau is the y value at infinite times. *K* is the rate constant, and *x* is the time. From equation (2), the initial rate equals – *K · y_0_* and the initial slope *s_i_* corrected for bleaching calculates as

(3) $$s_{i}=-K\cdot y_{0}-m$$

### EAE models

Active EAE was induced in 6-12 week old WT Lewis rats by subcutaneous immunization with MBP in CFA as described above (Generation and culturing of T_MBP_ cells).

Passive transfer EAE was induced in 6-12 week old WT Lewis rats by intravenous injection of 5 × 10^6^ fully activated T_MBP_ cell blasts (day 2 after *in vitro* stimulation).

Weight and clinical scores were recorded daily. EAE symptoms were scored as follows: 0, no disease; 1, flaccid tail; 2, gait disturbance; 3, complete hind limb paralysis; 4, tetraparesis; 5, death. No statistical method was used to predetermine sample size, experiments were not randomized, and investigators were not blinded to treatment group allocation.

### *In vivo* administration of the drugs

The animals were treated daily with 2ME2, its derivative STX140, or the STS inhibitor Irosustat starting from the day of EAE induction. The compounds were dissolved in a vehicle, namely 40 % 2-hydroxypropyl-β-cyclodextrin (PanReac AppliChem). Vehicle treatment alone served as control. The animals were injected intraperitoneally with 50 μL / 100 g body weight of the indicated compounds and doses. The i.p. route was chosen although, unlike 2MeE2, both STX140 and Irosustat possess good oral bioavailability, in order to have an effective drug comparison.

### Cell isolation and flow cytometry

For the assessment of CNS immune cell infiltration, rats were sacrificed at the peak of the disease. Single cell suspensions were obtained from the spinal cord passing the tissue through a cell-strainer. Immune cells were then purified by using a 2-phase Percoll-density gradient and characterized by using the following anti-rat monoclonal antibodies: αβTCR-AF647 (clone R73, Biolegend), CD8α-PE (OX-8; Biolegend), CD4-PECy7 (clone OX-35, BD Biosciences), CD11b/c-AF647 (clone OX-42, Biolegend), MHC class II-FITC-(OX-6), CD45RA-PE (clone OX-33; Biolegend). Cytofluorometric quantification of T cells was performed by relating the number of cells to a known absolute number of fluorescent beads (CaliBRITE, BD Biosciences).

To assess T cell activation at the protein level in vitro, surface staining for CD25 (OX39, Bio-Rad) was performed. APC-labelled goat anti-mouse antibody (Jackson ImmunoResearch) was used as secondary antibody. Mouse IgG1κ (MOPC 31 C) was used as control. Note that the experiments were performed in parallel with experiments of a previously published study (Löhndorf *et al*., 2021). Therefore, the data of the control groups are the same here as in that publication.

Cytofluorometric analysis was carried out with a CytoFLEX S (Beckman Coulter) operated by CytExpert software (Beckman Coulter). All procedures were performed at 4 °C. Data were analysed by using FlowJo software (FlowJo, LLC).

### Quantitative pCR

Total RNA was isolated using the standard Trizol method. cDNA was synthesized using the RevertAid First Strand cDNA Synthesis Kit (Thermo Fisher Scientific). Quantitative PCR (qPCR) was performed using the StepOnePlus™ Real-Time PCR System (Applied Biosystems), as previously described (Cordiglieri *et al*., 2010; Flügel *et al*., 2001; Odoardi *et al*., 2007). Rat T_MBP_ cell data were obtained from three to four independent duplicate measurements. β-actin served as a housekeeping gene. Human PBMC data were obtained from two independent healthy donors and duplicate measurements (representative data of one donor are shown). HPRT served as a housekeeping gene. For all probes (TaqMan), a combination of 5’-FAM and 3'-TAMRA quencher was used. Note that the experiments were performed in parallel with experiments of a previously published study (Löhndorf *et al*., 2021). Therefore, the data of the control groups are the same here as in that publication.

### Statistics

Data analysis was performed with Excel (Microsoft Office Standard 2016) and Prism (Version 9, GraphPad Software). Normal distribution was investigated by Kolmogorov-Smirnov test with Dallal-Wilkinson-Lillie for *p* value. Normally distributed data were analysed by unpaired two-tailed *t* test or ANOVA and Bonferroni correction for multiple testing with a significance level of α = 0.05. Non-normally distributed data or data with a small sample size was analysed by non-parametric Kruskal-Wallis test with Dunn’s correction for multiple testing also with a significance level of α = 0.05. An *a priori* power analysis to estimate the sample size was not performed.

## Results

### STX140 inhibits SOCE in the human Jurkat T cell line and in primary rat T cells

**Figure 2:**
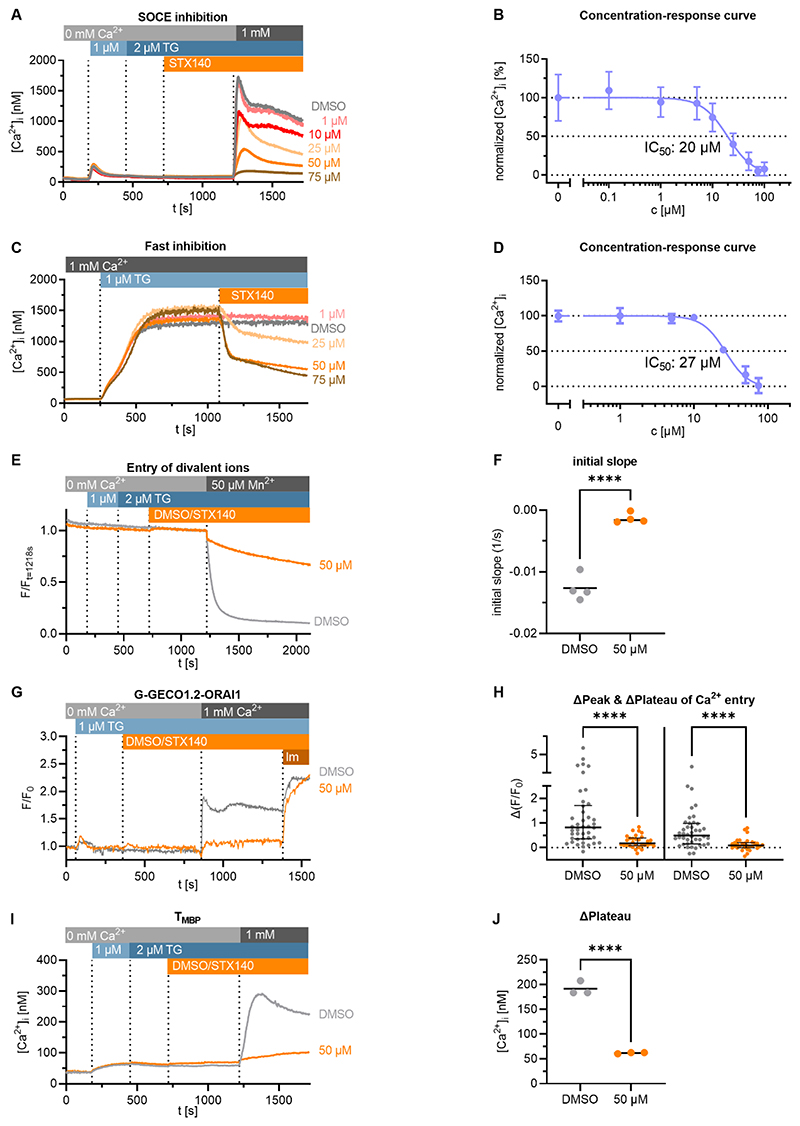
STX140 inhibits SOCE in human Jurkat T cells and encephalitogenic primary rat T cells. **A**: Mean Ca^2+^ tracings of Jurkat T cells treated with increasing concentrations of STX140 or vehicle (0.2 % DMSO). Representative data of 4 independent measurements. **B**: Concentration-response curve of STX140 of the Δplateau Ca^2+^ concentrations (mean ± S.D.). The calculated IC50 value is 20 μM. **C**: Mean tracings of Ca^2+^ signals evoked by 1 μM thapsigargin (TG) in presence of the indicated concentrations of STX140 or 0.2 % DMSO. Representative data of 3 independent experiments. **D**: Concentration-response curve of STX140 of the Δplateau Ca^2+^ concentrations divided by the ΔPlateau Ca^2+^ concentrations after thapsigargin stimulation (mean ± SD). The calculated IC50 value is 27 μM. **E-F**: SOCE evoked Mn^2+^ quenching of Fura2 in Jurkat T cells is prevented by 50 μM STX140. The decrease of Fura2 fluorescence after Mn^2+^ addition was fitted to an exponential one-phase decay equation (colored lines, **E**). The slope of this decay was subtracted by the slope of a straight line fitted to the data before Mn^2+^ addition and is displayed in **F**. Dots and lines depict individual experiments and mean values, respectively. Representative data of 4 independent experiments. ****: p < 0.0001. Unpaired two-tailed *t* test. **G-H:** SOCE in G-GECO1.2-ORAI1-transfected Jurkat T cells. **G**: Mean Ca^2+^ tracings of the different conditions. **H**: Inhibition of Δpeak (left) and Δplateau (right) values of SOCE at 50 μM STX140. Representative data of 4 independent experiments analyzing 42 (DMSO) or 30 (STX140) cells. Median ± IQR. ****: p < 0.0001. Kruskal-Wallis-test. **I-J**: Mean Ca^2+^ tracings and Δplateau values (mean) of rat T_MBP_ cells treated with 0.2 % DMSO or 50 μM STX140. Dots and lines depict individual experiments and mean values, respectively. Representative data of 3 independent experiments. ****: p < 0.0001. Unpaired two-tailed *t* test.

In a Ca^2^free/Ca^2+^ re-addition protocol performed on Jurkat T lymphocytes, STX140 inhibited SOCE upon thapsigargin stimulation in a concentration-dependent manner with an IC50 of about 20 μM (Fig. 2A,B). Furthermore, STX140 inhibited established Ca^2+^ entry in Ca^2+^ buffer immediately after compound addition with an IC50 of about 27 μM (Fig. 2C,D). In line with these data, Mn^2+^ quenching of Fura2 ratio was blocked by STX140 (Fig. 2E,F). In Jurkat T cells expressing G-GECO-Orai1.2 to sense Ca^2+^ ions at the mouth of the channel pore, highly significant inhibition by 50 μM STX140 of the initial and sustained Ca^2+^ entry phases were observed (Fig. 2G,H). In order to confirm the effects of STX140 in non-transformed cells, we repeated the measurements in primary rat T cells reactive for myelin basic protein (T_MBP_ cells), known to induce EAE. In these T cells, SOCE was also inhibited by 50 μM STX140 (Fig. 2I,J).

### STX140 inhibits T cell activation in vitro

**Figure 3:**
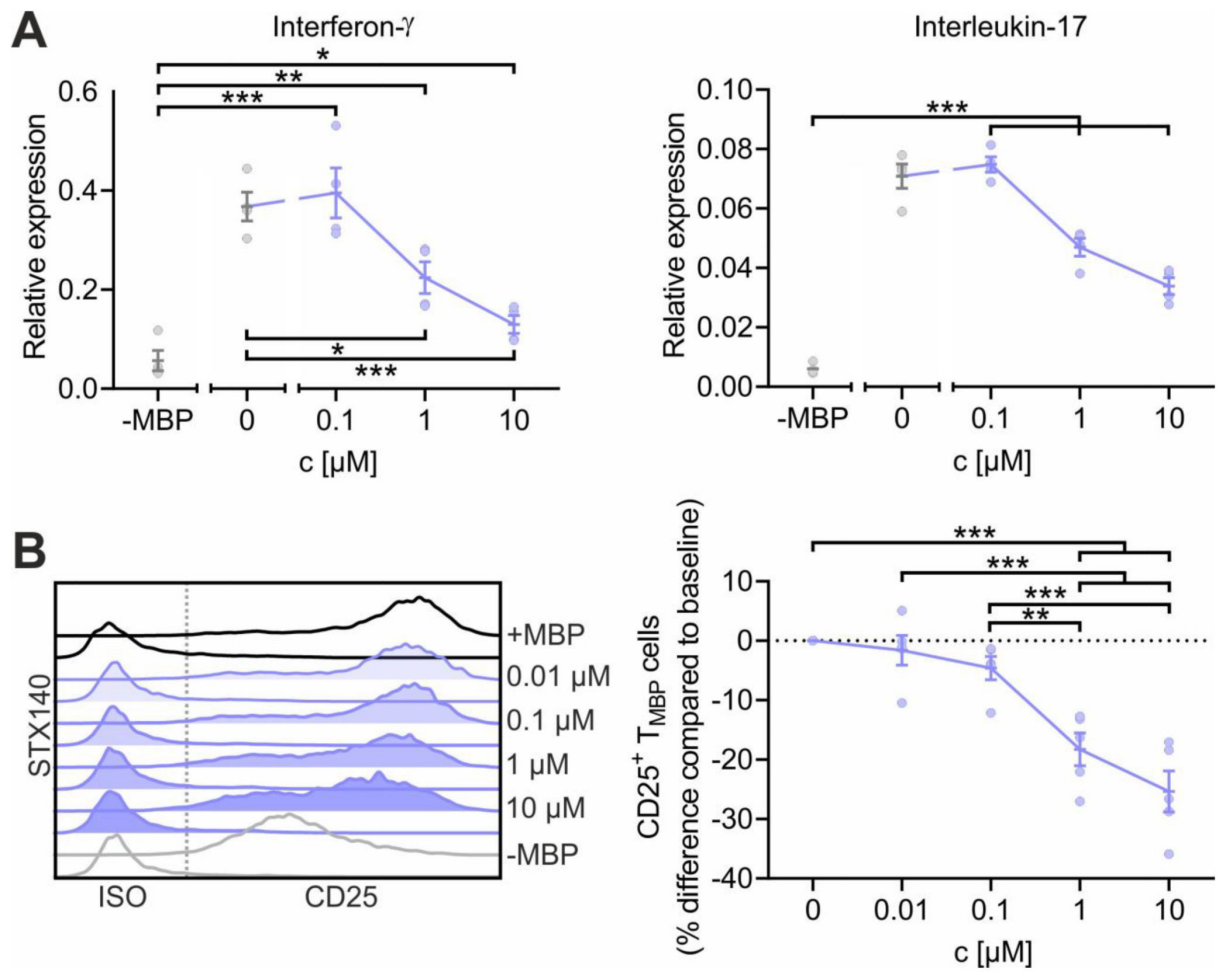
STX140 inhibits antigen-dependent T_MBP_ cell cytokine production and T cell activation. T_MBP_ cells were challenged in vitro by the cognate myelin-basic protein (MBP) in presence of STX140 or 0.2 % DMSO (+ or –MBP) at the indicated concentrations. **A:** Depicted is the relative expression of interferon-γ, and interleukin-17 six hours after antigen exposure measured by quantitative PCR. Housekeeping gene: ß-actin. Non-stimulated T cells (–MBP) were used as negative control. **B:** Depicted are the histograms of CD25 expression on T_MBP_ cells and the corresponding change in CD25 expression compared to baseline based on the geometric mean of the fluorescence intensity (MFI). Analysis was performed six hours after antigen exposure. Please note that the control groups are identical with already published data (Löhndorf *et al*., 2021) as the experiments were performed in parallel. Mean ± S.E.M. of 4 (A) or 5 (B) independent experiments; *: p < 0.05, **: p < 0.01, ***: p < 0.001. A: Unpaired two-tailed *t* test. B: One-way ANOVA with Bonferroni corrections.

Ca^2+^ entry upon T cell receptor engagement induces a chain of downstream events in T cells that determines the upregulation of surface proteins such as CD25 (IL-2 receptor alpha chain) and the production of pro-inflammatory cytokines such as IFNγ and IL-17. We aimed to evaluate the effect of STX140 on those events in TMBP cells upon antigen stimulation. Antigen receptor signaling-dependent cytokine production was significantly reduced by STX140 starting at a concentration of 1 μM (Fig. 3A). These effects of STX140 on TMBP cell activation were further corroborated by measuring the protein expression of CD25. Upregulation of CD25 following T_MBP_ cell stimulation was significantly reduced at a concentration of ≥1 μM (Fig. 3B).

### STX140, but not 2ME2, blocks active EAE

**Figure 4:**
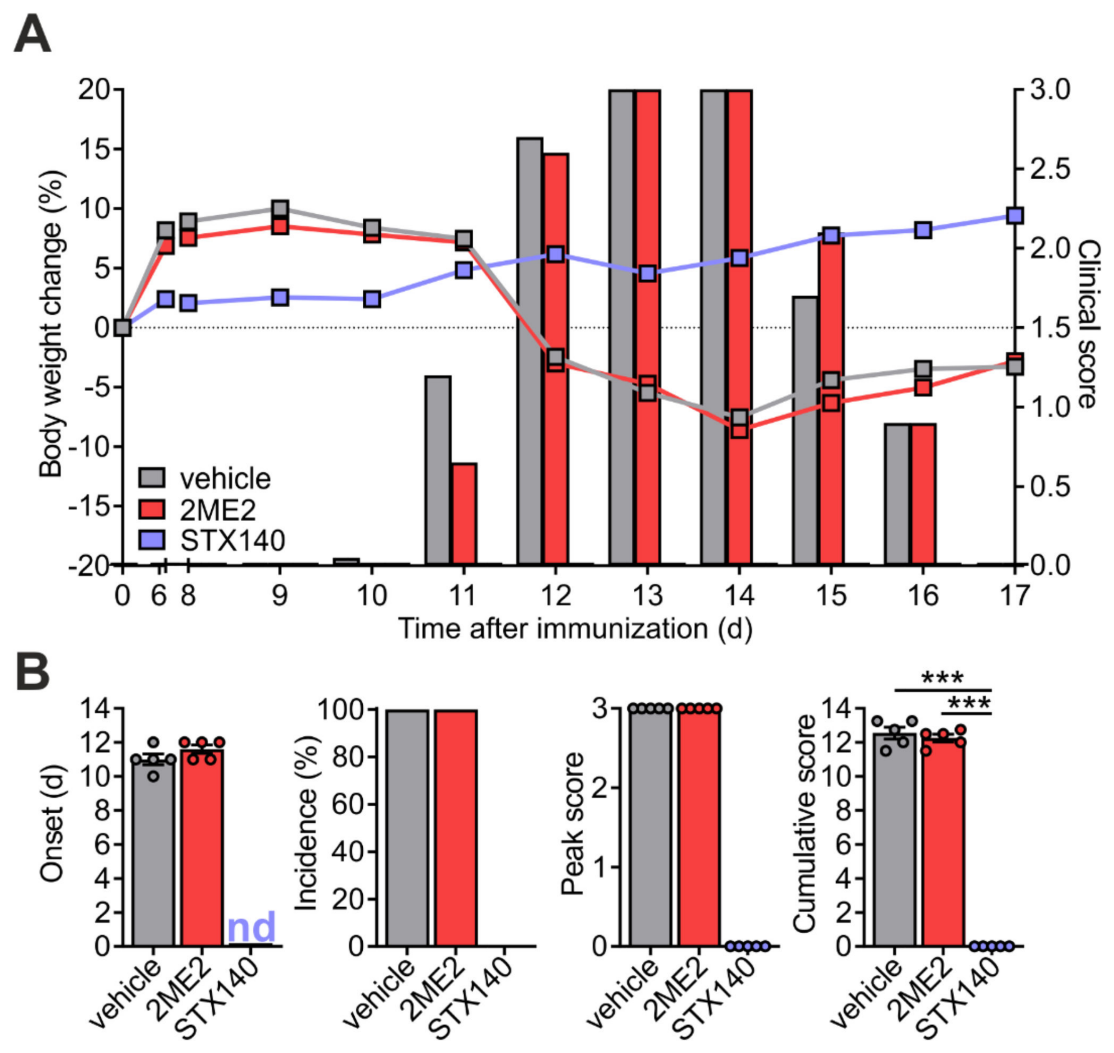
STX140 efficiently blocks active EAE. Active EAE was induced in Lewis rats by MBP immunization. **A and B**: Animals were treated i.p. with 2ME2, STX140 (both 10 mg/kg/day) or vehicle. **A**: Body weight change (lines) and clinical score (bars) measured daily. **B**: EAE onset, disease incidence, peak of clinical score and cumulative clinical score in vehicle- or drug-treated animals. Representative data of 2 independent experiments. Mean ± S.E.M; ***: p < 0.001. One-way ANOVA with Bonferroni corrections. nd = not determinable.

**Figure 5:**
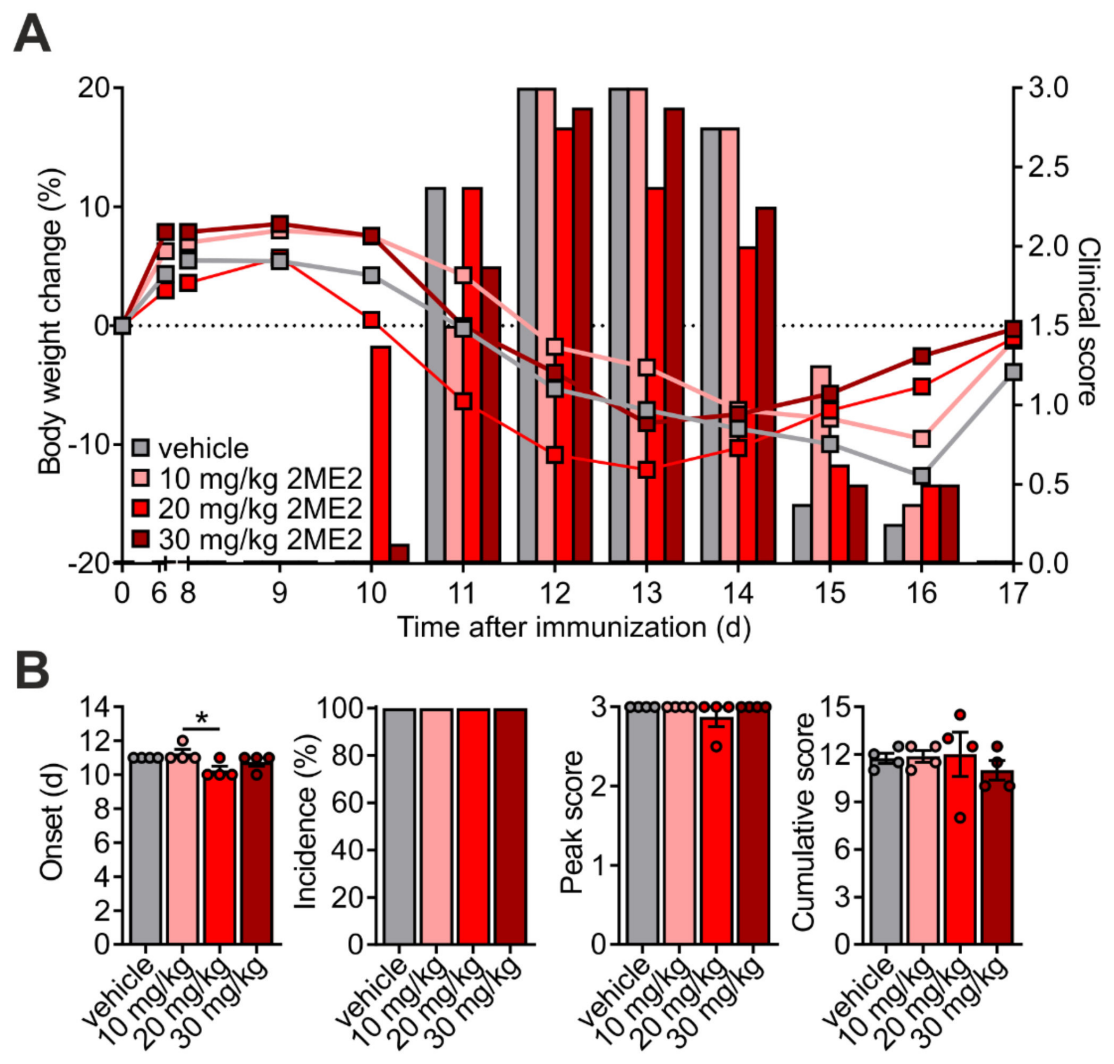
Even at higher doses, 2ME2 does not affect active EAE. Active EAE was induced in Lewis rats by MBP immunization. **A and B:** Animals were treated i.p. with 2ME2 at 10, 20 or 30 mg/kg/day. **A**: Body weight change (lines) and clinical score (bars) measured daily. **B**: EAE onset, disease incidence, peak of clinical score and cumulative clinical score in vehicle- or drug-treated animals. Representative data of 2 independent experiments. Mean ± S.E.M; *: p < 0.05. One-way ANOVA with Bonferroni corrections.

**Figure 6:**
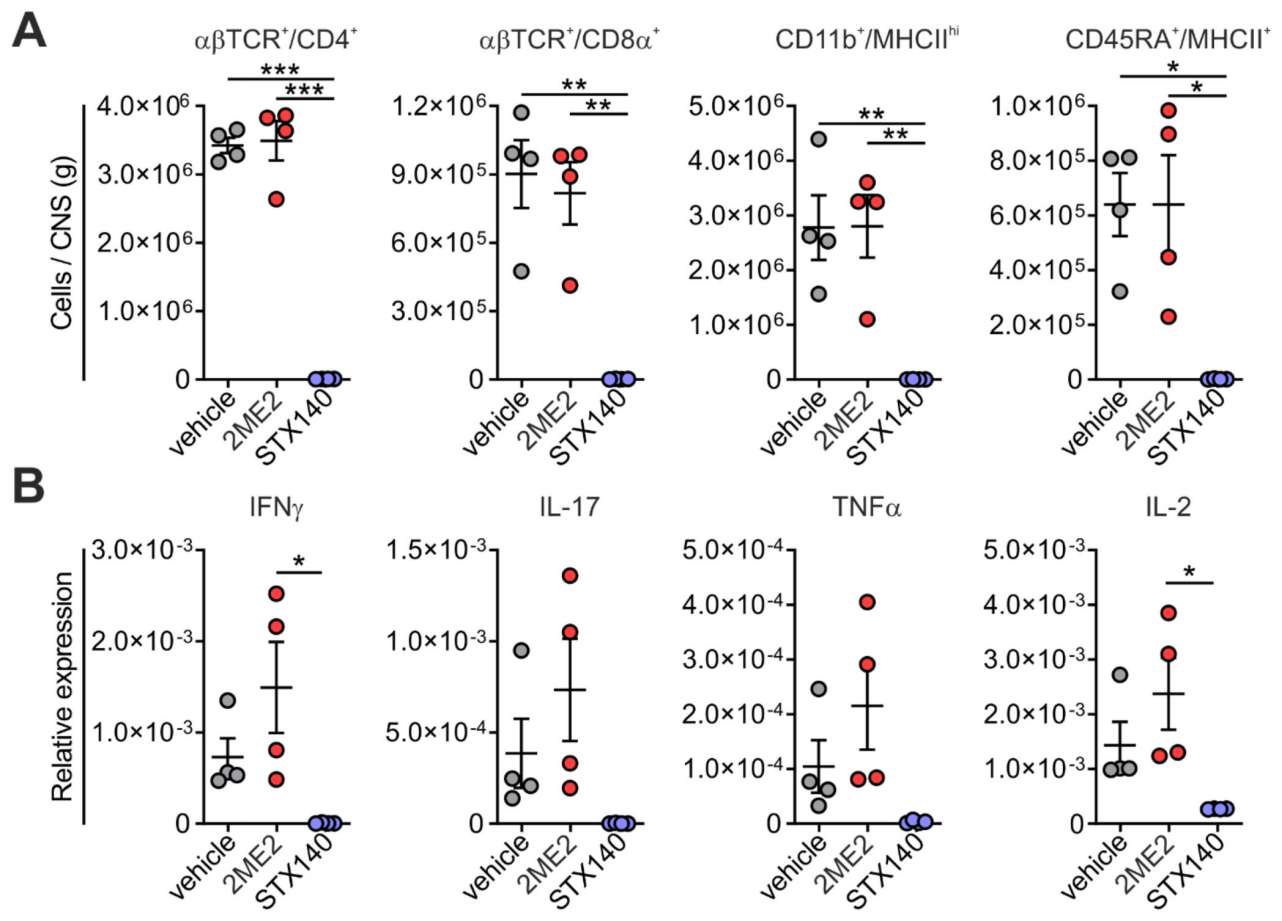
STX140 prevents immune cell infiltration into and inflammation of the CNS. Active EAE was induced in Lewis rats by MBP immunization. **A and B:** Animals were treated i.p. with 2ME2, STX140 (both 10 mg/kg/day) or vehicle. Immune cell infiltration and immune response in the CNS was analysed at the peak of the disease (clinical score 3 in the control group). **A**: Absolute number of the indicated immune cells in the spinal cord detected by flow cytometry. **B**: Corresponding expression of the indicated cytokines in total spinal cord tissue measured by quantitative PCR. House-keeping gene: β-actin. Mean ± S.E.M. from 4 animals/group; *: p < 0.05, **: p < 0.01, ***: p < 0.001. One-way ANOVA with Bonferroni corrections.

Having proved the efficacy of STX140 in blocking Ca^2+^ entry and T cell activation *in vitro*, we next investigated its effect *in vivo*. To this end, we employed a T cell-mediated CNS autoimmune disease, namely EAE in Lewis rats induced by active immunization with myelin antigen (active EAE). Active EAE evolves in two phases: in the initial disease-free phase, naïve T cells equipped with an appropriate antigen-specific T cell receptor become activated within the draining lymph nodes upon encounter of the myelin antigen presented by lymph node-resident antigen presenting cells (APCs). In the second phase, these now ‘primed’ cells migrate as effector T cells into the CNS where they re-encounter endogenous myelin antigen presented by brain-resident APCs. The ensuing re-activation and cytokine production of the autoaggressive effector T cells trigger a massive recruitment of immune cells into the CNS tissue. The resulting CNS inflammation induces neuronal dysfunction and consecutively a severe paralytic disease (Kawakami *et al*., 2004). Daily administration of STX140 i.p. completely prevented clinical symptoms at a dose of 10 mg/day (Fig. 4). In contrast to STX140, 2ME2 administered i.p. did not show any significant clinical effects, neither at 10 mg/day or at higher doses of up to 30 mg/day, which was the maximum concentration obtainable as a solution (Fig. 4 and 5). In line with the clinical data, STX140 but not 2ME2, prevented the infiltration of CD4^+^ and CD8^+^ T cells, CD11b^+^ myeloid cells and B cells into the CNS at a dose of 10 mg/kg/day (Fig. 6A). Accordingly, the inflammatory profile of the CNS tissue as indicated by the expression of the inflammatory cytokines IFNγ, IL-17, TNFα and IL-2 was significantly decreased in animals treated with STX140, but not when treated with 2ME2 (Fig. 6B).

### STX140, but not 2ME2, blocks adoptive transfer EAE

**Figure 7:**
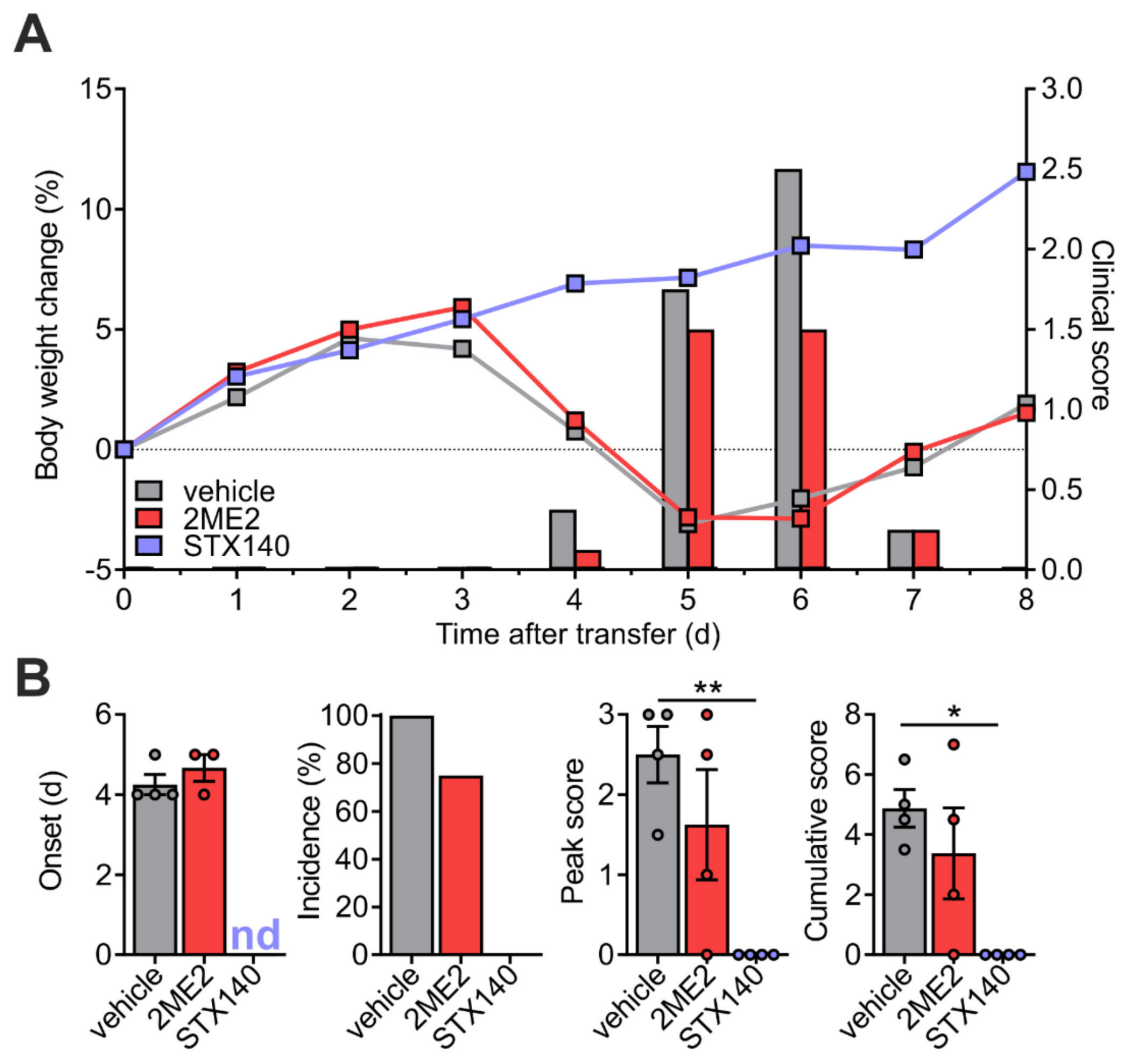
STX140 efficiently blocks transfer EAE. EAE was induced in Lewis rats by transfer of T_MBP_ cells. **A and B**: Animals were treated i.p. with 2ME2, STX140 (both 10 mg/kg/day) or vehicle. **A**: Body weight change (lines) and clinical score (bars) measured daily. **B**: EAE onset, disease incidence, peak of clinical score and cumulative clinical score in vehicle- or drug-treated animals. Representative data of 2 independent experiments. Mean ± S.E.M; *: p < 0.05; **: p < 0.01. One-way ANOVA with Bonferroni corrections. nd = not determinable.

Next, we tested the effect of STX140 on the re-activation of T_MBP_ cells in the CNS, the key step preceding CNS inflammation and paralytic disease. For this purpose, we used a model of EAE induced by adoptive transfer of autoreactive T cells. Notably, in this model, the initial T cell activation takes place *ex vivo* thus bypassing the induction phase within the lymph nodes. As observed for the active EAE, STX140 also completely prevented adoptive transfer EAE in rats at a dose of 10 mg/kg/day i.p. while 2ME2 did not have any effect (Fig. 7). Although, unlike 2MeE2, STX140 is known to have good oral bioavailability in order to have an effective *in vivo* comparison both drugs were dosed i.p.

### STX140 effect on inflammation does not depend on its STS activity

**Figure 8:**
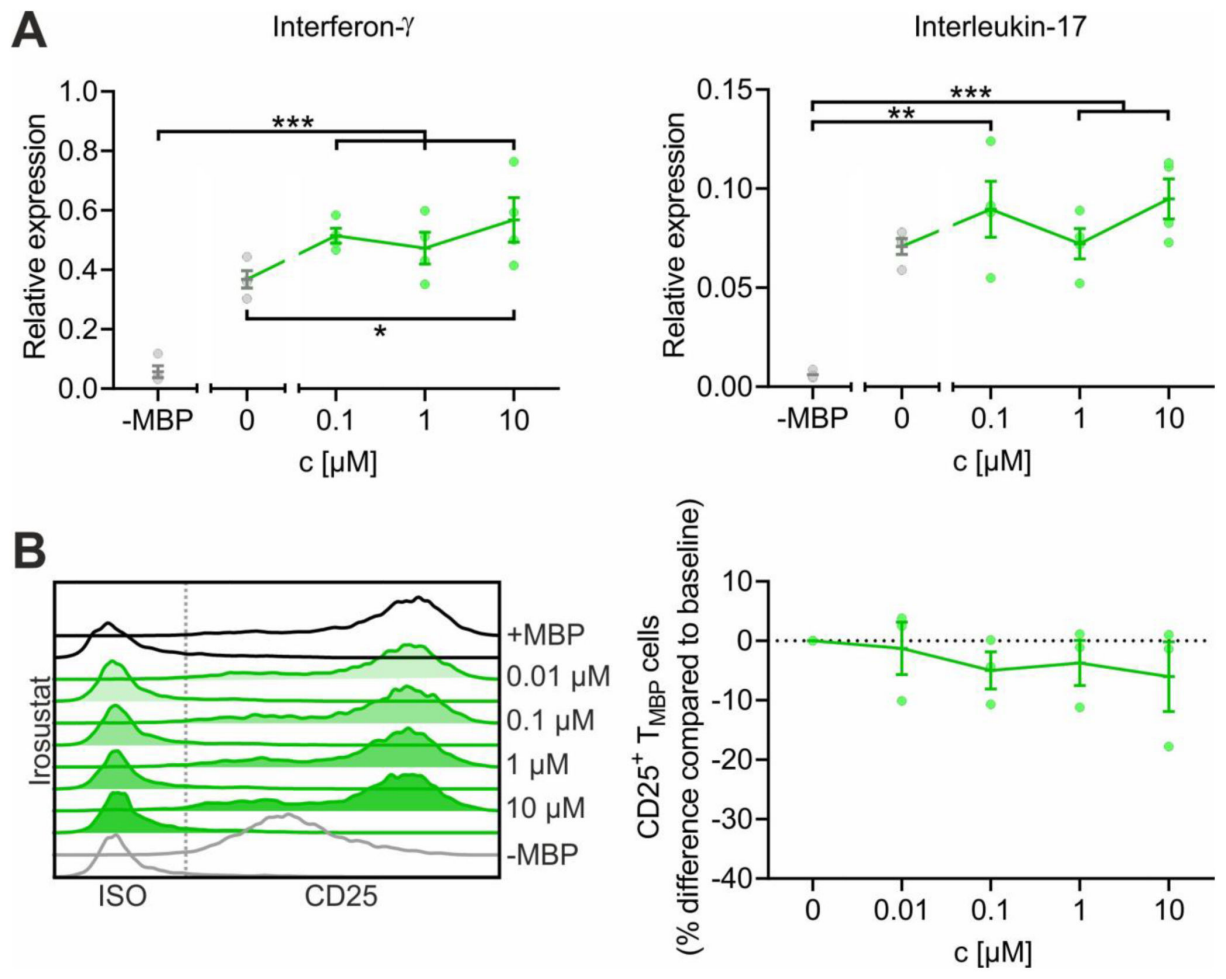
Irosustat does not inhibit antigen-dependent T_MBP_ cell cytokine production and T cell activation. T_MBP_ cells were challenged in vitro by the cognate myelin-basic protein (MBP) in presence of Irosustat or 0.2 % DMSO (+ or -MBP) at the indicated concentrations. **A:** Depicted is the relative expression of interferon-γ, and interleukin-17 six hours after antigen exposure measured by quantitative PCR. Housekeeping gene: β-actin. Non-stimulated T cells (-MBP) were used as negative control. **B:** Depicted are the histograms of CD25 expression on TMBP cells and the corresponding change in CD25 expression compared to baseline based on the geometric mean of the fluorescence intensity (MFI). Analysis was performed six hours after antigen exposure. Please note that the control groups are identical with already published data (Löhndorf *et al*., 2021) as the experiments were performed in parallel. Mean ± S.E.M. of 4 (A) or 3 (B) independent experiments; *: p < 0.05, **: p < 0.01, ***: p < 0.001. A: Unpaired two-tailed *t* test. B: One-way ANOVA with Bonferroni corrections.

**Figure 9:**
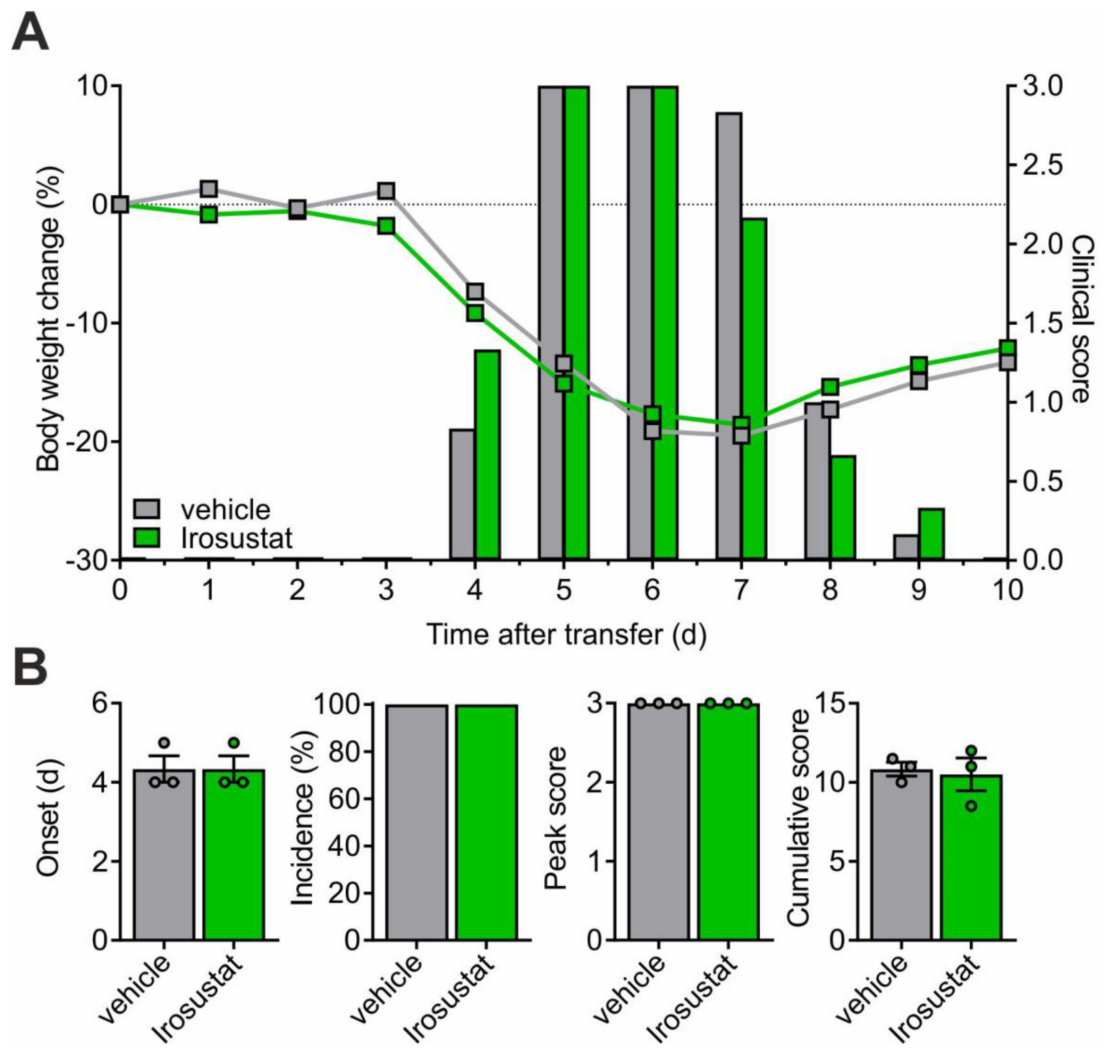
Irosustat does not affect transfer EAE. EAE was induced in Lewis rats by transfer of T_MBP_ cells. **A and B**: Animals were treated i.p. with Irosustat (10 mg/kg/day) or vehicle. **A**: Body weight change (lines) and clinical score (bars) measured daily. **B**: EAE onset, disease incidence, peak of clinical score and cumulative clinical score in vehicle- or drug-treated animals. Representative data of 2 independent experiments. Mean ± S.E.M. Unpaired two-tailed *t* test.

STX140, in addition to its other pharmacological activities, has a high STS inhibitory potency (39nM in placental microsomes) (Leese *et al*, 2006) that could potentially also contribute to its anti-inflammatory effect. To test this hypothesis, we examined the effects of the pure STS inhibitor Irosustat (STX64) on T cell mediated inflammation. In contrast to STX140 that significantly blocked CD25 upregulation and production of pro-inflammatory cytokines in T_MBP_ cells upon *in vitro* antigen stimulation (Fig. 3A,B), no such *in vitro* effects were observed in T_MBP_ cells stimulated in the presence of Irosustat/STX64 (Fig. 8A,B). In contrast to STX140, treatment with Irosustat/STX64 also did not have any clinical effect *in vivo* on EAE induced by transfer of T_MBP_ cells when dosed i.p. (Fig. 9A,B). Although Irosustat has excellent oral bioavailability, in order to ensure an effective *in vivo* comparison with STX140 both drugs were dosed i.p.

### STX140 inhibits interferon-γ expression by human PBMCs

**Figure 10:**
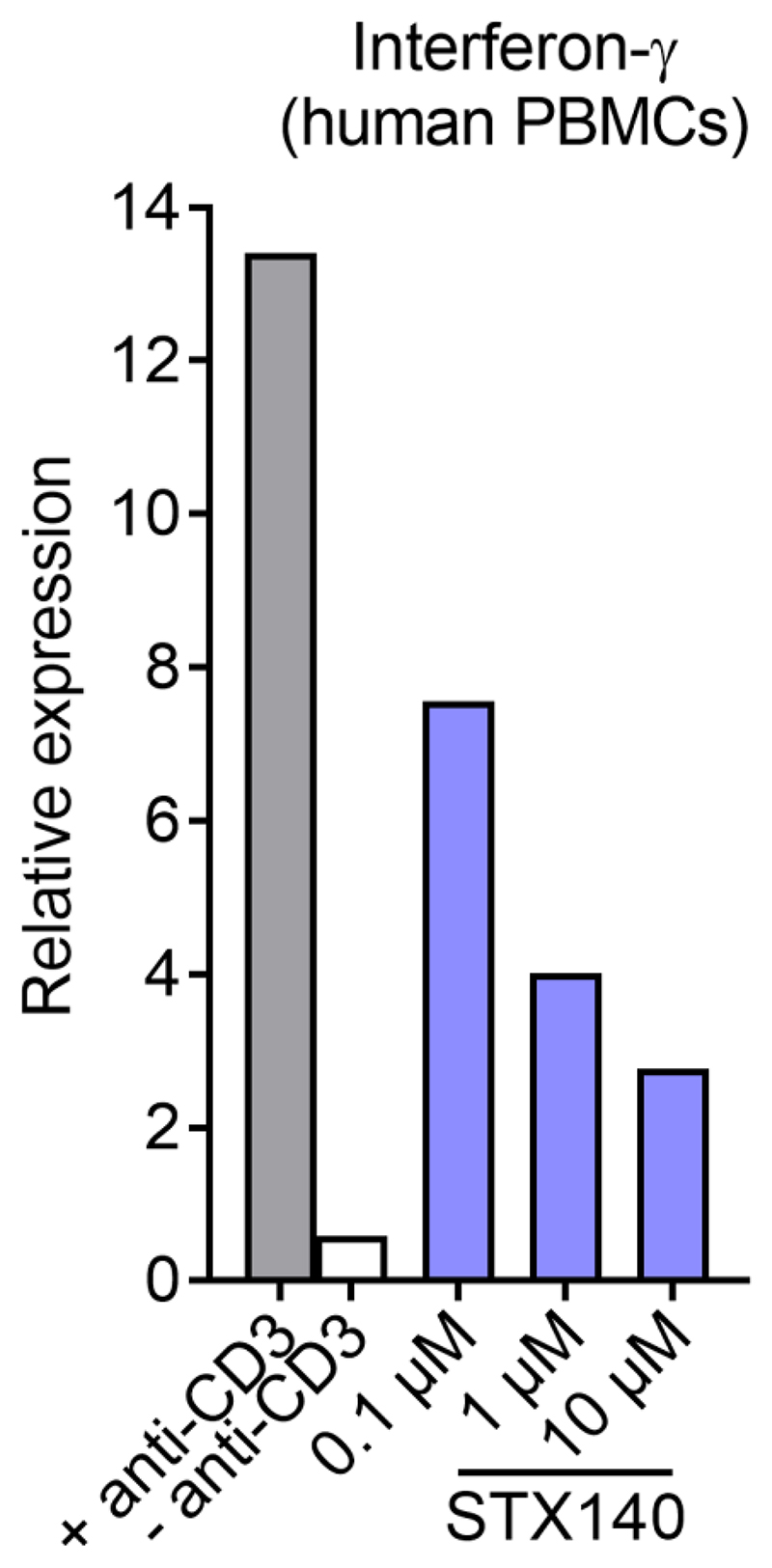
STX140 blocks IFNγ production in human PBMCs. Human PBMCs were challenged *in vitro* with anti-CD3 antibody in presence of STX140 at the indicated concentrations. CD3-stimulated (+ anti-CD3) or non-stimulated (–anti-CD3) PBMCs served as positive and negative control, respectively. Depicted is the relative expression of IFNγ24 h after stimulation measured by quantitative PCR. Housekeeping gene: HPRT. Representative data from 5 healthy donors.

Finally, we assessed the effect of STX140 on the activation of human PBMC. To this end IFNγ expression upon anti-CD3 stimulation was evaluated. In line with its effect on rat T cell activation, STX140 also inhibited IFNγ expression by human PBMCs in a concentration-dependent manner (Fig. 10).

## Discussion

Autoimmune diseases are initiated when autoreactive effector T cells invade their target organ and become locally re-activated upon encounter of their cognate antigen. The subsequent release of inflammatory mediators leads to the recruitment of additional immune cells and the onset of clinical disease (Kawakami *et al*., 2004; Becher *et al*., 2006; Odoardi *et al*., 2007; Bartholomäus *et al*., 2009). This sequence of events is proposed also to take place in MS where the misdirected T cells attack the CNS. The currently available therapeutic strategies directed to block this autoimmune attack are still unsatisfactory, although, admittedly, in the last decade considerable progress has been made concerning therapeutic options for the treatment of MS (Hartung & Kieseier, 2014). Several new drugs have been licensed such as dimethyl fumarate, fingolimod, alemtuzumab, teriflunomide and ocrelizumab. Still, many of these drugs have considerable side effects, preventing their use in all patients, so that the search for new compounds suitable to target other pathways in the pathogenic process is ongoing.

The drugs currently available are designed to interfere with different steps of the disease pathogenesis, e.g. the migration of T cells from lymphatic tissues into blood circulation (S1PR1 antagonists) or the transgression of the blood-brain-barrier (anti-VLA4 therapy). Here we explored another potential target, namely the activation of autoreactive T cells initiated by SOCE upon T cell receptor engagement. Interfering with Ca^2+^ signaling as an early and essential component of T cell activation might represent an alternative therapeutic approach.

A promising candidate drug in this context is 2-methoxyestradiol (2ME2). 2ME2, a well-known nonpolar and non-estrogenic, endogenous metabolite of estradiol, has been demonstrated to block T cell activation efficiently via the inhibition of SOCE *in vitro* (Löhndorf *et al*., 2021) and downstream events like NFAT translocation and cytokine production (Duncan *et al*., 2012; Löhndorf *et al*., 2021). Importantly, treatment with 2ME2 was also effective in the treatment of the MS animal model EAE: daily treatment with 100 mg completely blocked the development of the paralytic disease (Duncan *et al*., 2012), proving in principle that blocking T cell activation by interfering with SOCE may indeed represent a possible therapeutic strategy, but *vide infra*.

A major disadvantage of 2ME2 as a drug candidate is its very low oral bioavailability that could be slightly increased when the drug was formulated as a NanoCrystal dispersion (Panzem NCD, Tevaarwerk *et al* 2009). The poor bioavailability of 2ME2 is most likely due to its low aqueous solubility and extensive ‘first pass’ hepatic metabolism with conjugation of its free hydroxyl groups and oxidation. Indeed, metabolism studies showed that 80-95 % of the drug was oxidized to inactive metabolite and furthermore 80-90 % of both species were present as inactive glucuronide or sulfate conjugates (Verenich & Gerk, 2010). 2ME2 has nevertheless entered extensive human clinical trials (up to phase II) in oncology over the last two decades, achieving positive results and displaying a very safe profile (Ireson et al., 2004b; Sutherland et al., 2007; Potter, 2018). However, the adverse drug metabolism and pharmacokinetic (DMPK) properties constituted a significant barrier to a broad application of 2ME2 in clinical settings.

In order to overcome these disadvantages, a development program for compounds with an improved metabolic profile and better bioavailability was conducted. One of them, 2-methoxyoestradiol-3,17-*O,O*-bis-sulfamate (STX140; Leese *et al*., 2006) exhibited promising properties: It possesses an oral bioavailability of 85 %, low metabolism and showed very convincing efficacy in the inhibition of cell proliferation, tumour growth, neovascularization, and induction of apoptosis in various cancer models (Newman *et al*., 2007; Newman *et al*., 2008; Foster *et al*., 2008; Chander *et al*., 2007). Moreover, no metabolites of STX140 were detected in plasma after oral or intravenous dosing, with significant concentrations still detectable in plasma after its oral or intravenous administration even 24 h after oral dosing, showing that this drug is resistant to metabolism (Ireson *et al* 2004b). Additionally, STX140 like Irosustat and presumably other sulfamate-based drugs, is sequestered almost completely after oral dosing *in vivo* into red blood cells and this allows it to avoid first pass metabolism. In red blood cells it is bound to carbonic anhydrase II through a sulfamate moiety (Cozier *et al*. 2010). How it emerges subsequently to exert its activity in target tissues is still not completely clear, however, but this may possibly be enabled by equilibrium effects in areas of tumour hypoxia. Based on these findings and especially given its attractive high oral bioavailability, we hypothesized that STX140 might also represent a promising candidate drug for the inhibition of SOCE, with consequent anti-inflammatory properties in the context of EAE treament and MS therapy.

Indeed, STX140 efficiently inhibited SOCE in Jurkat T cells, as well as CNS autoimmune disease triggering T_MBP_ cells. Furthermore, it inhibited the activation of both T_MBP_ cells and human PBMCs in a concentration-dependent manner. While 2ME2 and STX140 displayed the same IC50 value (20 μM) and thus the same potency to inhibit SOCE, STX140 completely blocked active EAE at a daily dose of 10 mg/kg which corresponds to a dose about 10-fold lower than described for 2ME2 (Duncan *et al*., 2012). In line with the clinical data, also the immune cell infiltration and the inflammatory response of the CNS tissue were drastically reduced in the animals treated with STX140 but not 2ME2.

Remarkably, STX140 did not only block active, but also adoptive, transfer EAE in which the initial activation step in the periphery is bypassed because it was performed *in vitro* and only the second step of T cell reactivation in the CNS takes place *in vivo*. Therefore, inhibition of SOCE by STX140 also interferes with this therapeutically relevant step.

The route of drug administration is important generally for overall drug DMPK properties and toxicity. Although the drugs were administered i.p. and not p.o. it has been noted that administration of such small molecules results in faster and more complete absorption compared to the p.o. route (Al Shoyaib *et al*. 2020). However, importantly, the metabolic fate of i.p. administered drugs is still generally deemed to resemble that of orally administered agents, including propensity to first-pass metabolism, and thus the lack of activity of 2ME2 can presumably be ascribed to the metabolic oxidation and conjugation for this molecule as discussed earlier and the known much better comparative profile for STX140 vs 2ME2 via the oral route should be preserved in i.p. administration. There seems also no reason to presume that STX140 by this route is not also taken up almost completely into red blood cells as via p.o. administration and this may also contribute positively to its efficacy.

The administration of the STS inhibitor Irosustat/STX64 did not give rise to any activity against transfer EAE when dosed i.p.. Normally, this drug is dosed p.o. and has proven very efficacious in animal models of hormone-dependent cancer and in human clinical trial (Stanway *et al*. 2006; Potter 2018). Again, there is no reason to presume that this drug when given i.p. is also not similarly sequestered into red blood cells as previously noted (Ireson et al., 2004a) and is fully able to exert its known STS-inhibitory activity in this present model. The fact that no activity was noted both *in vitro* or *in vivo* importantly implies that there is no role for STS in this pathology and consequently that the potent activity of STX140 shown is a function of the whole molecule and is not a result of its additional potent STS inhibitory property. This is also important in terms of the previously observed anti-inflammatory activity of STS inhibitors, as discussed. Thus, the activity of the core 2ME2 structure (Duncan *et al*. 2012) is importantly preserved in STX140 (and possibly further enhanced), and bis-sulfamoylation of 2ME2, as expected, also affords better overall pharmaceutical and metabolic properties.

STS inhibition by STX140 in the context of T cell activation was considered the most obvious additional effect that may have caused amelioration of EAE clinical score (see discussion above). However, further off-target effects of STX140 cannot be excluded. Recently, we analyzed such potential off-target effects of a compound closely related to STX140, namely STX564 (Löhndorf et al., 2021). STX564 neither affected IP3 mediated Ca^2+^ release from the ER in permeabilized T cells, nor was the refilling of Ca^2+^ stores by SERCA pumps inhibited (Löhndorf et al., 2021). Further, K^+^ channels regulate the amplitude of Ca^2+^ entry by counterbalancing the increased positive charge of Ca^2+^ ions entering the cytosol. Analysis of K_V_ channels did not result in any significant inhibition by STX564 (Löhndorf et al., 2021). Though these results do not fully exclude effects of STX140 on these targets, the data with STX564 at least render such off-target effects unlikely.

As a further caveat, in terms of potential wider off-target effects it must also be noted that, like 2ME2 itself, STX140 and its closely related sulfamate esters (Leese *et al*., 2006) when investigated in oncology settings also exhibit cytotoxic activity but much more potently, possessing antiproliferative, antiangiogenic, and pro-apoptopic properties with induction of G2-M cell-cycle arrest, thought to be also primarily as a result of their anti-tubulin activity (Leese *et al*., 2006; Newman *et al*., 2008). It was concluded that disruption of nuclear translocation of NFAT is *largely* responsible for 2ME2’s inhibitory effects on lymphocytes *in vitro* and on EAE progression *in vivo* (Duncan *et al*., 2012) and the present and earlier study (Löhndorf *et al*., 2021) has now developed this further mechanistically with demonstration of SOCE inhibition for both drugs. Beyond this SOCE inhibition, however, it is unknown whether the cytotoxic activities of STX140 might also contribute to the potent *in vivo* effects on EAE observed and this is worthy of further investigation.

High serum 2ME2 concentrations following oral administration are difficult to achieve and it was postulated that 2ME2 concentrations in local tissue areas might therefore be significantly higher than those observed in serum (Duncan *et al*, 2012). This could also be important for the efficacy of STX140 *in vivo*, albeit differently as, unlike 2ME2, the drug is sequestered almost completely in red blood cells with its mechanism of eventual unloading in target tissue for oncology applications still unclear. It seems feasible in principle that targeted drug delivery might occur in areas of local inflammation by mechanisms yet to be clarified, but which could be relevant to the present study and is also worthy of future attention.

In summary, we have shown that STX140 suppresses inflammation and clinical symptoms in T cell-mediated CNS autoimmunity likely also largely by blocking SOCE and its downstream events of T cell activation. Due to its safe profile and particularly its high oral bioavailability, STX140 may be developed as a new therapeutic option for the treatment of MS and of other T cell-mediated autoimmune diseases. Given the impressive complete blockade of clinical disease in both EAE models at 10 mg/kg/day, to pursue such potential further it will be important in future studies to explore activity at even lower drug doses, as well as through dosing by the oral route.

## Data Availability

Source data are available from the corresponding authors upon request. Samples of STX140 and Irosustat are available from BVLP.
